# Safety and clinical outcomes of remdesivir in hospitalised COVID-19 patients: a retrospective analysis of active surveillance database

**DOI:** 10.1186/s12879-021-07004-8

**Published:** 2022-01-04

**Authors:** Vaishali Gupte, Rashmi Hegde, Sandesh Sawant, Kabil Kalathingal, Sonali Jadhav, Rohit Malabade, Jaideep Gogtay

**Affiliations:** 1 Medical Services, Cipla Ltd., Mumbai, India; 2 Head Clinical Research, Cipla Ltd., Mumbai, India; 3 Drug Safety, Cipla Ltd., Mumbai, India; 4 Medical Services, Clinical Trial Group, Cipla Ltd., Mumbai, India; 5 Global Chief Medical Officer, Cipla Ltd., Mumbai, India

**Keywords:** Remdesivir, COVID-19, Retrospective studies, Retrospective analysis, Active surveillance

## Abstract

**Background:**

Real-world data on safety and clinical outcomes of remdesivir in COVID-19 management is scant. We present findings of data analysis conducted for assessing the safety and clinical outcomes of remdesivir treatment for COVID-19 in India.

**Methods:**

This retrospective analysis used data from an active surveillance programme database of hospitalised patients with COVID-19 who were receiving remdesivir.

**Results:**

Of the 2329 patients included, 67.40% were men. Diabetes (29.69%) and hypertension (20.33%) were the most common comorbidities. At remdesivir initiation, 2272 (97.55%) patients were receiving oxygen therapy. Remdesivir was administered for 5 days in 65.38% of patients. Antibiotics (64.90%) and steroids (47.90%) were the most common concomitant medications. Remdesivir was overall well tolerated, and total 119 adverse events were reported; most common were nausea and vomiting in 45.40% and increased liver enzymes in 14.28% patients. 84% of patients were cured/improved, 6.77% died and 9.16% showed no improvement in their clinical status at data collection. Subgroup analyses showed that the mortality rate was significantly lower in patients < 60 years old than in those > 60 years old. Amongst patients on oxygen therapy, the cure/improvement rate was significantly higher in those receiving standard low-flow oxygen than in those receiving mechanical ventilation, non-invasive ventilation, or high-flow oxygen. Factors that were associated with higher mortality were age > 60 years, cardiac disease, diabetes high flow oxygen, non-invasive ventilation and mechanical ventilation.

**Conclusion:**

Our analysis showed that remdesivir is well tolerated and has an acceptable safety profile. The clinical outcome of cure/improvement was 84%, with a higher improvement in patients < 60 years old and on standard low-flow oxygen.

**Supplementary Information:**

The online version contains supplementary material available at 10.1186/s12879-021-07004-8.

## Introduction

Coronavirus disease 2019 (COVID-19) is a novel respiratory disease caused by severe acute respiratory syndrome coronavirus-2 (SARS-CoV-2). In March 2020, the COVID-19 outbreak was declared as a global pandemic by the World Health Organization (WHO) [1]. As of 28 December 2020, the global incidence of COVID-19 had reached 80, 838, 931 confirmed cases; India was the second most-affected country in the world, with a case burden of 10, 207, 871 confirmed cases [2]. As the pathogenesis underlying COVID-19 became more apparent, global strategies evaluating therapeutic options, including new antivirals, evolved rapidly. However, repositioning the already existing therapeutics remained a commonly adapted strategy recommended by the WHO [3].

Remdesivir is an adenosine analogue with broad-spectrum antiviral activity against several single-stranded RNA viruses. It was originally developed for treating patients with Ebola virus infection [4]. After recording the potential benefits of remdesivir against SARS-CoV-2 in in vitro, pre-clinical, and human cell line studies, its efficacy was evaluated in patients with COVID-19 [5–7]. On 1 May 2020, remdesivir received the Emergency Use Authorisation (EUA) status based on a preliminary report from an interim analysis of an ongoing double-blind randomised controlled trial by the United States Food Drug Administration (US FDA) [8]. On 21 June 2020, the Central Drugs Standard Control Organisation (CDSCO) approved its restricted emergency use for treating patients with severe COVID-19 infection in India; the indication was later expanded to moderate and severe disease. However, the CDSCO approved remdesivir with a condition to provide data from an active surveillance programme on a monthly basis by the pharmaceutical manufacturers [8]. Given the global emergency and the unmet medical need with respect to COVID-19 treatment, the Drugs Controller General of India (DCGI) also provided a clinical trial waiver for remdesivir use in India [8]. At the time of writing this paper, clinical evidence for its safety and efficacy in COVID-19 pertains mainly to randomised trials, and only few observational data are available that show its safety in real practice. In this paper, we present a retrospective analysis of data from an active surveillance programme conducted for remdesivir use in patients with COVID-19 in India.

## Methods

### Surveillance design and participants

This retrospective analysis evaluated active surveillance data of hospitalised patients with COVID-19 who received remdesivir treatment (Cipremi^®^; Cipla Ltd). Remdesivir was administered to patients with COVID-19 at participating hospitals in accordance with the restricted emergency use approval for remdesivir by CDSCO, India.

We retrospectively analysed the data of patients who had received remdesivir therapy from 09 July 2020 until 15 October 2020, with an aim to evaluate its safety and efficacy. All hospitals that administered remdesivir in COVID-19 patients were obliged to provide data through an active surveillance form.

### Procedure and outcomes

The physician/clinical staff filled an online surveillance form (Additional file 1: Fig. S1) for each patient with suspected or confirmed COVID-19 who was administered remdesivir. Data regarding patient age, gender, comorbid conditions, concomitant medicines, status of oxygen supplementation, remdesivir treatment duration, and any adverse event were collected. Clinical outcomes were defined as cure (complete resolution of symptoms), improvement, no improvement, or death. The data collection instrument did not allow knowing the severity of disease. The categorization to mild/moderate /severe COVID-19 was not performed.

### Statistical analysis

Continuous and quantitative variables are summarised using descriptive statistics. Categorical data are presented as frequency count (*N*) and percentages (%). All statistical analyses were performed using the Statistical Package for the Social Sciences version 23.0. A subgroup analysis was performed to assess the association of clinical and demographic characteristics with the clinical outcomes. Patients with missing information for a given variable were excluded from the calculations/analysis. A *p* value of < 0.05 was considered statistically significant.

## Results

### Patient demographics

Data of 2329 patients were available through the online or paper-based active surveillance log from 09 July 2020 up to 15 October 2020. The geographical distribution showed that Tamil Nadu (29.40%), Telangana (11.10%), Uttar Pradesh (10.00%), West Bengal (9.30%), and Maharashtra (8.80%) contributed maximally to the current data. Most patients were in the age group of 40–60 years (49.90%) followed by the age group of > 60 years (33.70%). Men comprised 67.40% of the analysed patient population (Table 1).

**Table 1:** Patient demographics

Characteristics	*N* (%)
Age group (years)	
< 12	2 (0.10)
≥ 12 to < 20	5 (0.20)
≥ 20 to < 40	376 (16.10)
≥ 40 to < 60	1162 (49.90)
≥ 60	784 (33.70)
Gender	
Male	1570 (67.40)
Female	583 (25.00)
Gender not disclosed	176 (7.60)
Comorbidities	
Diabetes	679 (29.69)
Hypertension	465 (20.33)
Cardiac disease	145 (6.34)
Lung disease	100 (4.37)
Need for oxygen supplementation	
SLFO	1483 (65.27)
HFO	433 (19.06)
NIV	273 (12.02)
MV	80 (3.52)
Concomitant medications	
Antibiotics	702 (64.90)
Steroids	518 (47.90)
Anticoagulants	345 (31.90)
Vitamin C	246 (22.80)
Zinc	124 (11.50)

*SLFO* standard low-flow oxygen, *HFO* high-flow oxygen, *NIV* non-invasive ventilation, *MV* mechanical ventilation

### Clinical characteristics

Up to 98.20% of patients had comorbid conditions. Diabetes (29.69%) was the most common comorbid condition followed by hypertension (20.33%), cardiac diseases (6.34%), and lung disease (4.37%) (Table 1). In addition to these comorbidities, 44.82% had other diseases such as blood cancer, cholangitis, and chronic kidney disease. A total of 2272 (97.55%) patients were receiving oxygen therapy at the time of starting remdesivir: the most common oxygen supplementation method was standard low-flow oxygen (65.27%), followed by high-flow oxygen (19.06%), non-invasive ventilation (12.02%), and mechanical ventilation (3.52%) (Table 1). Duration of remdesivir treatment administered in 65.38% of patients was 5 days, and was < 5 days in 22.52% of patients while only 12.11% of patients received remdesivir for ≥ 6 days. Among 1081 patients on concomitant medications, antibiotics were the most common concomitant medications (64.90% of the patients) followed by steroids (47.90% of the patients) (Table 1).

### Adverse events and safety

A total of 119 adverse events were reported. Most common were nausea and vomiting (45.40%) followed by increased liver enzyme levels (increased serum glutamic pyruvic transaminase [SGPT], serum glutamic oxaloacetic transaminase [SGOT] levels) (14.28%), rash (5.80%), bradycardia (2.50%), nephrotoxicity (1.70%), and oral ulcer (0.80%).

### Clinical outcomes

**Fig. 1:**
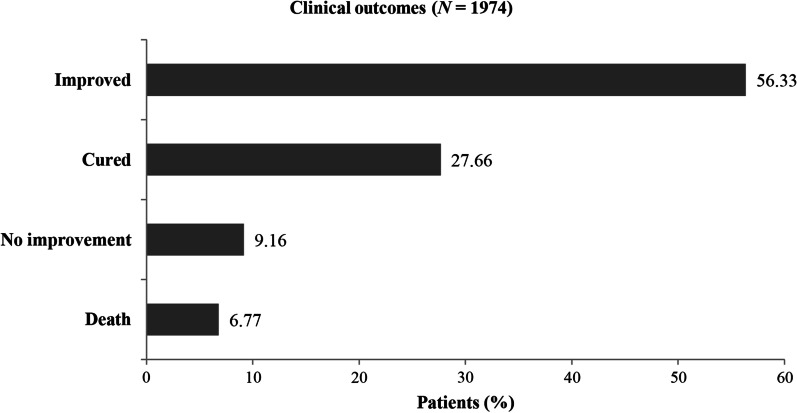
Clinical outcomes

Information regarding clinical outcome was available only for 1974 patients and it was missing for 355 patients. The clinical outcome of cure or improvement was recorded in 83.99% (improved, 56.33%; cured, 27.66%), death in 6.77% and no improvement was seen in 9.16% of the patients at the time of data collection (Fig. 1).

### Subgroup analysis

Results of various subgroup analyses are presented in Table 2. When clinical outcomes were analysed by patient age, cure/improvement rate was significantly higher in the age groups of 20–40 years (91.45%, *p* < 0.0001) and 40–60 years (85.33%, *p* = 0.0011) compared with that in the age group of ≥ 60 years (78.99%). Similarly, the cure/improvement rate was significantly higher in the age group of 20–40 years compared with that in the age group of 40–60 years (91.45% vs. 85.33%, *p* = 0.0083). Mortality rate was higher in patients ≥ 60 years old compared with those 20–40 years old (10.07% vs. 3.62%, *p* < 0.0001) and 40–60 years old (10.07% vs. 5.37%, *p* = 0.0004).

**Table 2:** Subgroup analysis of clinical outcomes by clinical and demographic characteristics

Characteristics	*N* (%)		
	Cure/improvement	Death/death related to COVID-19	No improvement
Age group (years)			
12–20	4 (80.00)	0 (0.00)	1 (20.00)
20–40	278 (91.45) ^#*^	11 (3.62) ^##**^	15 (4.93) ^###***^
40–60	826 (85.33) ^$^	52 (5.37) ^$$^	90 (9.30) ^$$$^
≥ 60	549 (78.99)	70 (10.07)	75 (10.79)
Gender			
Male	1,206 (83.29) ^@^	108 (7.46) ^@^	134 (9.25) ^@^
Female	451 (86.07)	26 (4.96)	47 (8.97)
Diabetes			
Yes	489 (78.36)	62 (9.93)	72 (11.54)
No	1,136 (85.52)	71 (5.41)	106 (8.07)
Hypertension			
Yes	377 (87.06)	23 (5.32)	33 (7.62)
No	1,248 (82.98)	110 (7.31)	145 (9.64)
Other cardiac conditions			
Yes	105 (81.40)	19 (14.73)	5 (3.88)
No	1,520 (84.07)	114 (6.31)	173 (9.57)
Lung disease			
Yes	72 (77.42)	10 (10.75)	11 (11.83)
No	1553 (84.22)	123 (6.67)	167 (9.06)
Received oxygen support			
Yes	1608 (83.62)	134 (6.97)	181 (9.41)
No	37 (100.00)	0 (0.00)	0 (0.00)
SLFO	1107 (93.97)	26 (2.21)	45 (3.82)
HFO	332 (80.58) ^§§^	24 (5.82) ^§^	56 (13.59) ^§§^
NIV	146 (57.71) ^§§^	48 (18.97) ^§§^	59 (23.32) ^§§^
MV	23 (28.75) ^§§^	36 (45.00) ^§§^	21 (26.25) ^§§^
Remdesivir treatment time			
< 5 days	312 (75.5) ^§§§^	59 (14.3) ^§§§^	42 (10.2)
5 days	1095 (87.0)	56 (4.4)	108 (8.6)
6–10 days	183 (85.9)^@@@^	14(6.6)^@@@^	16 (7.5)
> 10 days	15 (100)	0	0
Received steroids	383 (79.95) ^@@^	40 (8.35) ^@@^	56 (11.69) ^@@^
Received antibiotics	456 (79.31)	52 (9.05)	67 (11.65)
Received anticoagulants	253 (81.35)	19 (6.11)	39 (12.54)

The patient status “No improvement” in the last column was reported at the time of data entry which can eventually change

*SLFO* standard low-flow oxygen, *HFO* high-flow oxygen, *NIV* non-invasive ventilation, *MV* mechanical ventilation

^#^*p* = 0.0083 vs. 40–60 years; *p < 0.0001 vs. > 60 years; ^$^*p* = 0.0011 vs. > 60 years; ^##^*p* = 0.2812 vs. 40–60 years; **p < 0.0001 vs. > 60 years; ^$$^*p* = 0.0004 vs. > 60 years; ^###^*p* = 0.0219 vs. 40–60 years; ***p = 0.004 vs. > 60 years; ^$$$^*p* = 0.3515 vs. > 60 years; ^§^*p* = 0.001 vs. SLFO; ^§§^*p* = 0.0001 vs. SLFO; ^@^*p* = 0.156 for cure, 0.065 for death, and 0.916 for no improvement; ^@@^ *p* = 0.112 compared with patients not receiving steroids; ^§§§^p < 0.0001 vs. 5 days; ^@@@^p = 0.004 for cure/improvement and 0.007 for death/death related to COVID-19

Although the mortality rate was slightly higher among the older adults (> 60 years), difference in the mortality rate remained statistically non-significant for the age groups of 40–60 years and 20–40 years (5.37% vs. 3.62%; *p* < 0.2812). The clinical outcomes were not different between men and women (*p* > 0.05). Similar cure/improvement rates were observed irrespective of comorbid conditions (diabetes: 78.36%, hypertension: 87.06%, cardiac diseases: 81.40% and lung disease: 77.42%). Mortality rate was significantly lower among those who received standard low-flow oxygen (2.21%) compared with those who received mechanical ventilation (45%, *p* < 0.0001), non-invasive ventilation (18.97%, *p* < 0.0001), or high-flow oxygen (5.82%, *p* < 0.001). Interestingly, mortality rate was not different between patients who received and those who did not receive concomitant steroids (*p* = 0.112).

A multivariate analysis was performed to assess the association of clinical and demographic characteristics with the clinical outcomes (Table 3). Results of multivariate analysis indicated that comorbidities such as cardiac disease and diabetes increased the odds of death by 3.76 and 1.55 times compared with absence of these comorbidities, respectively. Similarly, the odds of death were higher amongst patients receiving high-flow oxygen, non-invasive ventilation, or mechanical ventilation compared with standard low-flow oxygen.

**Table 3:** Multivariate analysis

Variables	Odds ratio	95% Confidence interval	p-value	Adjusted odds ratio	95% Confidence interval	p-value
Age group (years)						
20–40	1.00					
40–60	1.51	(0.78, 2.94)	0.222			
≥ 60	2.99	(1.56, 5.73)	0.001			
Gender						
Male	1.54	(0.99, 2.40)	0.062			
Female	1.00					
Co-morbidities						
Hypertension (Yes/No)	0.71	(0.45, 1.13)	0.146			
Diabetes (Yes/No)	1.93	(1.36, 2.75)	0.000	1.55	(1 01, 2.39)	0.05
Lung disease (Yes/No)	1.85	(0.96, 3.56)	0.063			
Cardiac (Yes/No)	2.57	(1.52, 4.33)	0.000	3.76	(1.95, 7.25)	< 0.001
Type of oxygen support						
SLFO	1.00					
HFO	2.74	(1.55, 4.83)	0.000	2.35	(1.26, 4.32)	0.007
NIV	10.37	(6.29, 17.10)	0.000	11.04	(6.4, 18.9)	< 0.001
MV	36.25	(20.14, 65.23)	0.000	40.51	(21.010, 77.7)	< 0.001
Remdesivir treatment duration						
< 5 days	1					
5 days	0.28	(0.19, 0.41)	< 0.001	0.29	(0.19, 0.45)	< 0.001
≥ 6 days	0.39	(0.21, 0.72)	0.002	0.40	(0.20, 0.80)	0.009
Concomitant medications						
Steroids	1					
Others	1.08	(0.69, 1.69)	0.735			

*SLFO* standard low-flow oxygen, *HFO* high-flow oxygen, *NIV* non-invasive ventilation, *MV* mechanical ventilation

Since there is no date of symptom onset, we also did a subgroup analysis to evaluate effectiveness of remdesivir based on date of hospitalization. The results indicated that there was a significant difference in clinical improvement rates when the treatment initiation < 3 days vs. other groups (Additional file 2: Tables S1 to S4). Analysis after removing the patients with suspected COVID-19, results showed that there was not much difference in outcome between COVID-19 laboratory-confirmed cases and total cases (Additional file 2: Table S5 and S6).

## Discussion

In this retrospective analysis of data from an active surveillance programme, we assessed safety of remdesivir by measuring the clinical outcomes (cure, improvement, no improvement, or death) in hospitalised patients with COVID-19 who were treated with remdesivir, and their subgroups. The cure/improvement rate was 84%, which is in accordance with the cure/improvement reported in historical cohorts [9–16]. Most patients were male, were in the age group of 40–60 years, required oxygen therapy, and received remdesivir for 5 days. Diabetes and hypertension were the most common comorbidities. Low cure rate and high mortality was seen in patients > 60 years old and in patients who required HFO, NIV or MV compared to patients on SLFO supplementation.

Remdesivir is a nucleotide prodrug effective against various RNA viruses such as Nipah virus, respiratory syncytial virus, Ebola virus, SARS-CoV-1, and the Middle East respiratory syndrome coronavirus (MERS-CoV) [17]. Remdesivir is metabolised intracellularly to its ATP analogue, which inhibits viral RNA polymerase and thus halts viral replication [4]. Remdesivir emerged as a candidate drug in the current COVID-19 pandemic and received full approval for use in COVID-19 by the USFDA [4, 18]. Remdesivir was well tolerated in clinical trials and in the compassionate-use programme. Common adverse events reported were nausea, elevated ALT levels, headache, hypokalaemia, worsening respiratory failure, and constipation [9–11, 13, 14]. In our analysis, 13% patients reported adverse events, with nausea and increased liver enzyme levels being the most common.

In the randomised, controlled ACTT-1 trials, patients in the remdesivir group were significantly more likely to have clinical improvements than those in the placebo group [9, 11]. In a robust pooled analysis that included patients enrolled in the SIMPLE-severe trial and a retrospective cohort of severe COVID-19 patients receiving standard-of-care, recovery rate was 74.4% at day 14 for patients in the remdesivir group [12]. An improvement in the clinical status by at least 2 points on the ordinal scale (or being discharged alive) was seen in 71.9% of the patients and that by ≥ 1 points was seen in 76.2% of the patients on day 14 [12]. The cure/improvement rate in our analysis was 83.99%.

In the SIMPLE-severe trial, patients randomised to a 5- or 10-day course of remdesivir did not show a significant difference [10]. Results from a recent DISCOVERY trial also showed no clinical benefit of remdesivir in hospitalised COVID-19 patients who were symptomatic for more than 7 days and receiving oxygen support [19]. Our analysis showed that most patients received a 5-day course of remdesivir therapy, which is in line with the DCGI-approved prescribing information.

The mortality rate following 5 days of treatment with remdesivir was 8% on day 14 in the SIMPLE-severe trial and 1% on day 28 in the SIMPLE-moderate study [9, 10]. Following 10 days of treatment with remdesivir, the mortality rate by day 15 was 6.7% in the ACTT-1 trial and 2% by day 14 in the SIMPLE-severe trial [10, 11]. The WHO Solidarity trial, which included 11,330 patients from 30 countries, showed no improvement in the mortality rate in patients randomised to remdesivir treatment compared with the local standard-of-control [20]. In another pooled analysis, the mortality rate was 7.6% at day 14 in patients receiving remdesivir for 5–10 days [12]. Two recent systematic review and meta-analysis have also supported the mortality benefits with the treatment of remdesivir [21, 22]. In our analysis, the mortality rate was 6.77% and multivariate analysis showed that remdesivir treatment for > 5 days was associated with lower odds of death compared to remdesivir treatment for < 5 days.

During the COVID-19 pandemic, several studies reported that older adults and those with comorbid hypertension, diabetes, obesity, and heart disease are at higher risk for developing life-threatening COVID-19 illness [14, 23]. In the current analysis, similar cure/improvement rate was observed irrespective of comorbid conditions. However, numerically higher mortality was observed in patients with cardiac disease (14.73%), followed by that in lung disease (10.75%), diabetes (9.93%), and hypertension (5.32%). Lower grade of respiratory support and age < 65 years were associated with a > 2 point improvement on the ordinal scale in patients treated with remdesivir [24]. Our results showed higher patient clinical outcome when patients did not require oxygen support and were < 60 years of age. An Indian retrospective study, the SORT trial enrolled 350 patients treated with remdesivir and showed that patients who received remdesivir early (within 9 days of symptom onset) were more likely to have a lower incidence of mortality compared with those treated after ≥ 9 days of symptom onset, suggesting that initiating remdesivir earlier during the disease course in moderate-to-severe COVID-19 infection may show better clinical improvements/outcomes [16].

Our analysis had some limitations as well. This was a retrospective analysis of the data obtained from an active surveillance programme database; therefore, comparison of the results with the control group could not be performed. Moreover, this analysis could not detect the association using multivariate analysis. Cure/improvement rates were not defined in terms of the ordinal scale. The data collection instrument did not allow to collect information regarding the severity of disease and therefore it is most likely that majority of these patients were moderate or severe. Data was collected at a single time point. However, our analysis presents findings of a large cohort of COVID-19 patients treated with remdesivir in real-life clinical settings in India and adds to the clinical evidence on remdesivir use in COVID-19.

## Conclusion

The retrospective analysis of data from an active surveillance programme of remdesivir therapy in patients with COVID-19 showed that remdesivir was well tolerated and had an acceptable safety profile. The clinical outcome of cure and improvement rate was 84%, with greater improvement in patients with age < 60 years and receiving standard low-flow oxygen and mortality rate was 6.77%.

## Supplementary Information

**Additional file 1: Fig. S1** Patient’s active surveillance log.

**Additional file 2: Table S1.** Association between clinical outcome vs. number of days of remdesivir treatment (date of remdesivir start—date of hospital admission). **Table S2.** Association between Outcome vs. Remdesivir start duration (< 3 days vs. 3–5 days). **Table S3.** Association between Outcome vs. Remdesivir start duration (< 3 days vs. 6–7 days). **Table S4.** Association between Outcome vs. Remdesivir start duration (< 3 days vs. > 7 days). **Table S5.** Frequency of laboratory confirmed cases. **Table S6.** Clinical outcomes in cases with laboratory confirmed COVID-19.

## Data Availability

The raw datasets used and/or analysed during the current study are available from the corresponding author on reasonable request.

## Abbreviations

COVID-19: Coronavirus disease 2019
SARS-CoV-2: Severe acute respiratory syndrome coronavirus-2
WHO: World Health Organization
US FDA: United States Food Drug Administration
CDSCO: Central Drugs Standard Control Organisation
DCGI: Drugs Controller General of India
SGPT: Serum glutamic pyruvic transaminase
SGOT: Serum glutamic oxaloacetic transaminase
MERS-CoV: Middle East respiratory syndrome coronavirus

## References

[1] Cucinotta D, Vanelli M (2020). WHO declares COVID-19 a pandemic. Acta Biomed.

[2] WHO Coronavirus (COVID-19) Dashboard. URL: https://covid19.who.int/. Accessed 02 June 2021.

[3] Kupferschmidt K, Cohen J. WHO launches global megatrial of the four most promising coronavirus treatments. Science. 2020. https://www.sciencemag.org/news/2020/03/who-launches-global-megatrial-four-most-promising-coronavirus-treatments. Accessed 17 March 2021.

[4] Pardo J, Shukla AM, Chamarthi G, Gupte A (2020). The journey of remdesivir: from Ebola to COVID-19. Drugs Context..

[5] Wang M, Cao R, Zhang L, Yang X, Liu J, Xu M (2020). Remdesivir and chloroquine effectively inhibit the recently emerged novel coronavirus (2019-nCoV) in vitro. Cell Res.

[6] Williamson BN, Feldmann F, Schwarz B, Meade-White K, Porter DP, Schulz J (2020). Clinical benefit of remdesivir in rhesus macaques infected with SARS-CoV-2. bioRxiv..

[7] Pizzorno A, Padey B, Julien T, Trouillet-Assant S, Traversier A, Errazuriz-Cerda E (2020). Characterization and treatment of SARS-CoV-2 in nasal and bronchial human airway epithelia. bioRxiv..

[8] Singh D, Wasan H, Mathur A, Gupta YK (2020). Indian perspective of remdesivir: a promising COVID-19 drug. Indian J Pharmacol.

[9] Spinner CD, Gottlieb RL, Criner GJ, Arribas López JR, Cattelan AM, Soriano Viladomiu A (2020). Effect of remdesivir vs standard care on clinical status at 11 days in patients with moderate COVID-19: a randomized clinical trial. JAMA.

[10] Goldman JD, Lye DCB, Hui DS, Marks KM, Bruno R, Montejano R (2020). Remdesivir for 5 or 10 days in patients with severe Covid-19. N Engl J Med.

[11] Beigel JH, Tomashek KM, Dodd LE, Mehta AK, Zingman BS, Kalil AC (2020). Remdesivir for the treatment of Covid-19—preliminary report. N Engl J Med.

[12] Olender SA, Perez KK, Go AS, Balani B, Price-Haywood EG, Shah NS (2020). Remdesivir for severe COVID-19 versus a cohort receiving standard of care. Clin Infect Dis.

[13] Grein J, Ohmagari N, Shin D, Diaz G, Asperges E, Castagna A (2020). Compassionate use of remdesivir for patients with severe Covid-19. N Engl J Med.

[14] Zhou F, Yu T, Du R, Fan G, Liu Y, Liu Z (2020). Clinical course and risk factors for mortality of adult inpatients with COVID-19 in Wuhan, China: a retrospective cohort study. Lancet.

[15] Lapadula G, Bernasconi DP, Bellani G, Soria A, Rona R, Bombino M (2020). Remdesivir use in patients requiring mechanical ventilation due to COVID-19. Open Forum Infect Dis.

[16] Mehta RM, Bansal S, Bysani S, Kalpakam H (2020). A shorter symptom-onset to remdesivir treatment (SORT) interval is associated with a lower mortality in moderate-to-severe COVID-19: a real-world analysis. medRxiv..

[17] Singh AK, Singh A, Singh R, Misra A (2020). Remdesivir in COVID-19: a critical review of pharmacology, pre-clinical and clinical studies. Diabetes Metab Syndr.

[18] US Food and Drug Administration. FDA’s approval of Veklury (remdesivir) for the treatment of COVID-19—The Science of Safety and Effectiveness. October 22, 2020. https://www.fda.gov/drugs/drug-safety-and-availability/fdas-approval-veklury-remdesivir-treatment-covid-19-science-safety-and-effectiveness. Accessed 17 March 2021.

[19] Ader F, Bouscambert-Duchamp M, Hites M, Peiffer-Smadja N, Poissy J, Belhadi D (2021). Remdesivir plus standard of care versus standard of care alone for the treatment of patients admitted to hospital with COVID-19 (DisCoVeRy): a phase 3, randomised, controlled, open-label trial. Lancet Infect Dis.

[20] Pan H, Peto R, Henao-Restrepo AM, Preziosi MP, Sathiyamoorthy V, WHO Solidarity Trial Consortium (2021). Repurposed antiviral drugs for COVID-19—interim WHO Solidarity trial results. N Engl J Med.

[21] Elsawah HK, Elsokary MA, Abdallah MS, ElShafie AH (2021). Efficacy and safety of remdesivir in hospitalized Covid-19 patients: systematic review and meta-analysis including network meta-analysis. Rev Med Virol.

[22] Wilt TJ, Kaka AS, MacDonald R, Greer N, Obley A, Duan-Porter W (2021). Remdesivir for adults with COVID-19: a living systematic review for American college of physicians practice points. Ann Intern Med.

[23] Cummings MJ, Baldwin MR, Abrams D, Jacobson SD, Meyer BJ, Balough EM (2020). Epidemiology, clinical course, and outcomes of critically ill adults with COVID-19 in New York City: a prospective cohort study. Lancet.

[24] Simon Collins, HIV i-Base. Predictors of response to remdesivir in GS-5773 COVID-19 study. 2020. https://i-base.info/htb/38476. Accessed 17 March 2021.

