# Supplementary figures and images for: Safety and clinical outcomes of remdesivir in hospitalised COVID-19 patients: a retrospective analysis of active surveillance database
Source: BMC Infect Dis. 2022 Jan 4;22:1. doi: 10.1186/s12879-021-07004-8 (PMC8724590; doi:10.1186/s12879-021-07004-8)

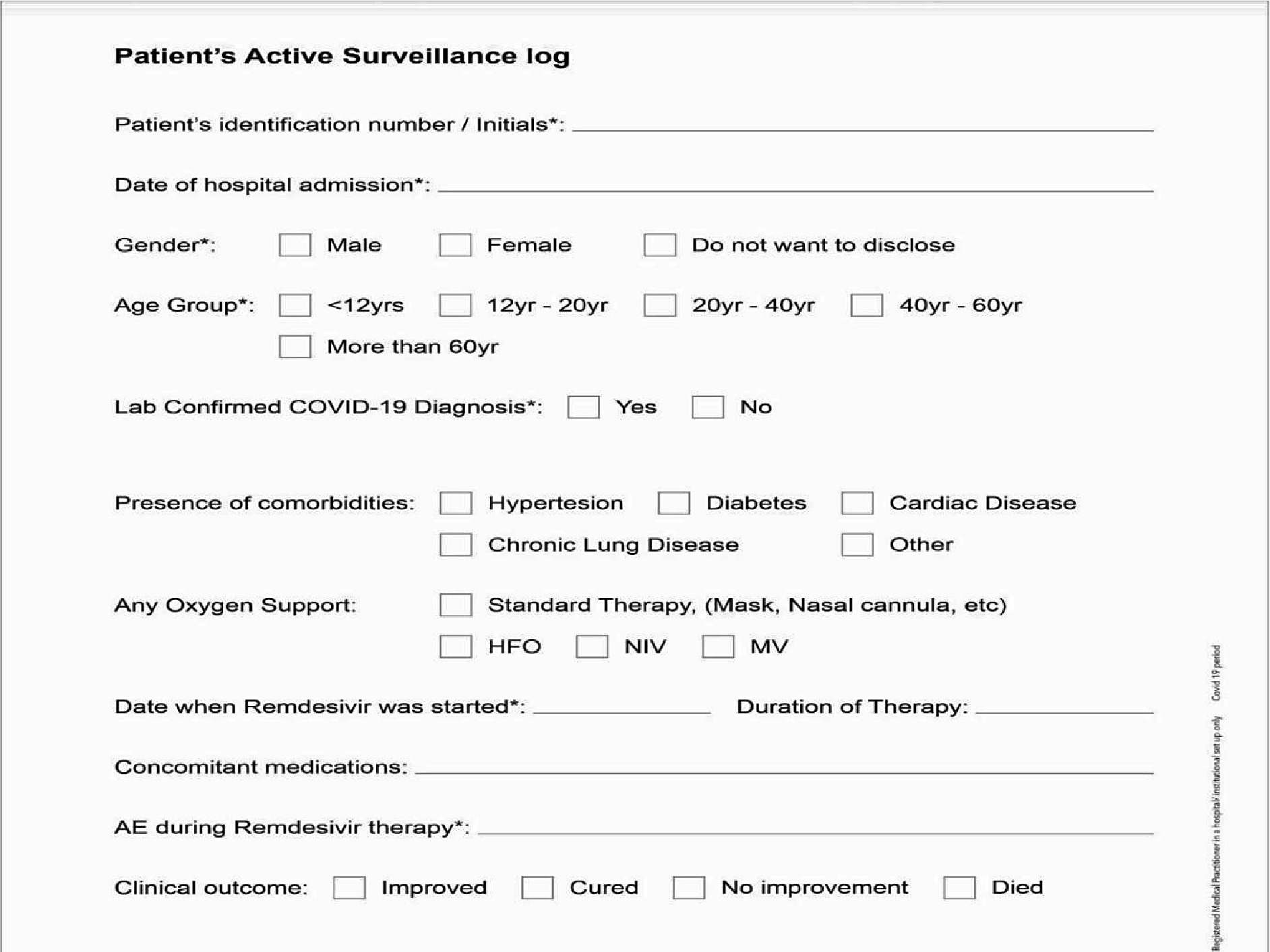

Supplement: Supplementary file 1 — Additional file 1: Fig. S1 Patient’s active surveillance log. [file 12879_2021_7004_MOESM1_ESM.tif]
