# Supplementary material for: Safety and clinical outcomes of remdesivir in hospitalised COVID-19 patients: a retrospective analysis of active surveillance database
Source: BMC Infect Dis. 2022 Jan 4;22:1. doi: 10.1186/s12879-021-07004-8 (PMC8724590; doi:10.1186/s12879-021-07004-8)
Supplement: Supplementary file 2 — Additional file 2: Table S1. Association between clinical outcome vs. number of days of remdesivir treatment (date of remdesivir start—date of hospital admission). Table S2. Association between Outcome vs. Remdesivir start duration (< 3 days vs. 3–5 days). Table S3. Association between Outcome vs. Remdesivir start duration (< 3 days vs. 6–7 days). Table S4. Association between Outcome vs. Remdesivir start duration (< 3 days vs. > 7 days). Table S5. Frequency of laboratory confirmed cases. Table S6. Clinical outcomes in cases with laboratory confirmed COVID-19. [file 12879_2021_7004_MOESM2_ESM.docx]

**Table S1. Association between clinical outcome Vs. number of days of remdesivir treatment (date of remdesivir start - date of hospital admission)**

| **Cured/Improved** | | **No. of Days (Date of Remdesivir start - Date of Hospital Admission)** | | | | **Total** | p-value |
| --- | --- | --- | --- | --- | --- | --- | --- |
|  |  | **< 3 days** | **3-5 days** | **6-7 days** | **>7 days** |  |  |
| Cured/Improved | Frequency | 1129 | 288 | 68 | 118 | 1603 | <0.001 |
|  | % | 86.4% | 80.7% | 73.9% | 76.6% | 84.0% |  |
| Death/Death related to COVID19 | Frequency | 77 | 32 | 7 | 13 | 129 |  |
|  | % | 5.9% | 9.0% | 7.6% | 8.4% | 6.8% |  |
| No Improvement | Frequency | 100 | 37 | 17 | 23 | 177 |  |
|  | % | 7.7% | 10.4% | 18.5% | 14.9% | 9.3% |  |
| Total | Frequency | 1306 | 357 | 92 | 154 | 1909 |  |
|  | % | 100.0% | 100.0% | 100.0% | 100.0% | 100.0% |  |

**Table S2. Association between Outcome Vs. Remdesivir start duration (<3 days Vs. 3-5 days)**

| **Outcome** | | (Date of Remdesivir start - Date of Hspital Admission) | | Total | p-value |
| --- | --- | --- | --- | --- | --- |
|  |  | < 3 days | 3-5 days |  |  |
| Cured/Improved | Frequency | 1129 | 288 | 1417 | 0.022 |
|  | % | 86.4% | 80.7% | 85.2% |  |
| Death/Death related to COVID19 | Frequency | 77 | 32 | 109 |  |
|  | % | 5.9% | 9.0% | 6.6% |  |
| No Improvement | Frequency | 100 | 37 | 137 |  |
|  | % | 7.7% | 10.4% | 8.2% |  |
|  | Frequency | 1306 | 357 | 1663 |  |
|  | % | 100.0% | 100.0% | 100.0% |  |

**Table S3. Association between Outcome Vs. Remdesivir start duration (<3 days Vs. 6-7 days)**

| **Outcome** | | Recode Days (Date of Remdesivir start - Date of Hspital Admission) | | Total | p-value |
| --- | --- | --- | --- | --- | --- |
|  |  | < 3 days | 6-7 days |  |  |
| Cured/Improved | Frequency | 1129 | 68 | 1197 | <0.001 |
|  | % | 86.4% | 73.9% | 85.6% |  |
| Death/Death related to COVID19 | Frequency | 77 | 7 | 84 |  |
|  | % | 5.9% | 7.6% | 6.0% |  |
| No Improvement | Frequency | 100 | 17 | 117 |  |
|  | % | 7.7% | 18.5% | 8.4% |  |
|  | Frequency | 1306 | 92 | 1398 |  |
|  | % | 100.0% | 100.0% | 100.0% |  |

**Table S4. Association between Outcome Vs. Remdesivir start duration (<3 days Vs. >7 days)**

| **Outcome** | | Recode Days (Date of Remdesivir start - Date of Hspital Admission) | | Total | p-value |
| --- | --- | --- | --- | --- | --- |
|  |  | < 3 days | >7 days |  |  |
| Cured/Improved | Frequency | 1129 | 118 | 1247 | 0.003 |
|  | % | 86.4% | 76.6% | 85.4% |  |
| Death/Death related to COVID19 | Frequency | 77 | 13 | 90 |  |
|  | % | 5.9% | 8.4% | 6.2% |  |
| No Improvement | Frequency | 100 | 23 | 123 |  |
|  | % | 7.7% | 14.9% | 8.4% |  |
|  | Frequency | 1306 | 154 | 1460 |  |
|  | % | 100.0% | 100.0% | 100.0% |  |

**Table S5. Frequency of laboratory confirmed cases**

| **Variables** | **Frequency** | **%** |
| --- | --- | --- |
| **Lab Confirmed COVID19 Diagnosis** | | |
| Yes | 2280 | 97.90% |
| No | 49 | 2.10% |
|  | 2329 |  |

**Table S6. Clinical outcomes in cases with laboratory confirmed COVID-19**

| **Clinical Outcome Code** | | **Lab Confirmed COVID19 Diagnosis** | | **Total** |
| --- | --- | --- | --- | --- |
|  |  | No | Yes |  |
| Cured/Improved | Freq | 31 | 1627 | 1658 |
|  | % within Lab Confirmed COVID19 Diagnosis | 64.6% | 84.5% | 84.0% |
| Death/Death related to COVID19 | Count | 7 | 127 | 134 |
|  | % within Lab Confirmed COVID19 Diagnosis | 14.6% | 6.6% | 6.8% |
| No Improvement | Count | 10 | 171 | 181 |
|  | % within Lab Confirmed COVID19 Diagnosis | 20.8% | 8.9% | 9.2% |
| Total | Count | 48 | 1925 | 1973 |
|  | % within Lab Confirmed COVID19 Diagnosis | 100.0% | 100.0% | 100.0% |
